# Malignant transformation of hyperplastic gastric polyps: An immunohistochemical and pathological study of the changes of neoplastic phenotype

**DOI:** 10.3892/ol.2014.1932

**Published:** 2014-03-04

**Authors:** JOHJI IMURA, SHINICHI HAYASHI, KAZUHITO ICHIKAWA, SHIGEHARU MIWA, TAKAHIKO NAKAJIMA, KAZUHIRO NOMOTO, KOICHI TSUNEYAMA, TATSUYA NOGAMI, HITOAKI SAITOH, TAKAHIRO FUJIMORI

**Affiliations:** 1 Department of Diagnostic Pathology, Graduate School of Medicine and Pharmaceutical Sciences, University of Toyama, Toyama 930-0194, Japan; 2 Department of Molecular and Surgical Pathology, Dokkyo Medical University School of Medicine, Mibu, Tochigi 321-0293, Japan; 3 Department of Pathology, Ibaraki Prefectural Central Hospital, Kasama, Ibaraki 309-1793, Japan

**Keywords:** gastric hyperplastic polyp, malignant transformation, clinicopathological study, mucin phenotype, tight junction, p53, proliferative activity

## Abstract

In spite of the evidence that the malignant transformation of gastric hyperplastic polyps (HPs) is a rare event, it must always be taken into account during diagnosis. The aim of the current study was to clarify the mechanism of the malignant transformation of gastric hyperplasia polyps, with focus on phenotypic expression, cell proliferation and p53 overexpression. Immunohistochemistry for mucin phenotypic markers, including MUC1, MUC2, MUC5AC, MUC6, tight junction factors (claudin-3, -4 and -18), an intestinal phenotypic marker [caudal type homeobox 2 (Cdx2)], Ki-67 proliferative index and p53 overexpression, was performed on archival specimens of gastric polyps excised from six patients. Histologically, the intermingled components of several lesions were present in these polyps. Furthermore, the cancer components were predominantly differentiated adenocarcinoma. Immunohistochemically, all hyperplastic components expressed MUC5AC, but did not exhibit positivity for MUC2. Additionally, the majority of hyperplastic components were immunonegative for claudin-3, while claudin-3 positivity was observed in the majority of areas of dysplasia and carcinoma. Expression of claudin-4 was also observed in the majority of cases and claudin-18 was preserved in the hyperplastic, dysplastic and adenocarcinomatous lesions of all cases. Nuclear accumulation of Cdx2 was detected in almost all the samples with dysplasia and carcinoma, while nuclear p53 was detected in 24–80% of the dysplastic areas and >85% of the cancer components. The Ki-67 labeling index appeared to correlate with neoplastic progression. The observations provided evidence that the mechanism underlying malignant transformation of gastric HPs may occur by multistep carcinogenesis, such as the hyperplasia-adenoma (dysplasia)-adenocarcinoma sequence, and these neoplastic cells may acquire various phenotypes during this process.

## Introduction

Hyperplastic polyps (HPs) are the most common type of lesion among polypoid lesions of the stomach. The reported incidence of focal malignancy in these polypoid lesions differs significantly between 0.8 and 7.1%. Although, HPs are considered to be relatively harmless in their natural course, a general understanding which has gained acceptance following a number of previous follow-up studies (1–4). However, certain authors have previously reported a few cases of the malignant transformation of gastric HPs (5–7); although, the incidence rate of malignant change has been reported to be relatively low, with an average of only ~2.1% in large series (6). With the recent prevalence of endoscopic treatments for gastric polyps, including polypectomy and mucosal resection, an increased number of cases of dysplasia or carcinoma arising in gastric HP have been reported (6,8–13). Previously, Yao *et al* proposed four characteristics of the malignantly transformed lesions associated with gastric hyperplasia polyps: i) lesions are predominantly of the well-differentiated type, although, a small number of lesions of the poorly differentiated type have also been reported; ii) malignant transformation has been suggested to be associated with dysplasia, although, involvement of intestinal metaplasia remains unknown; iii) the mucin phenotype appears to be of the gastric type in a number of cases; and iv) p53 may be important in the malignant transformation (14).

Previous histochemical and immunohistochemical analyses for mucins have indicated that differentiated adenocarcinomas may be classified into gastric and intestinal phenotypes as in Lauren’s classification (15). A number of markers have been reported to distinguish between gastric and intestinal mucins; however, a set of markers that are able to completely distinguish between the two mucin phenotypes has not yet been reported (16–19).

Tight, adherent and gap junctions, as well as desmosomes, are well-known cell membrane structures that are involved in cell-to-cell interactions. Adhesion tight junctions, present in epithelial and endothelial cell membranes, form a component of the intercellular junctional complexes and are important in barrier function, cell polarity and cell signaling pathways (20). Claudins are major tight junction constituents and exhibit four transmembrane domains. To date, 24 members of the claudin family have been identified (20). From this family of proteins, claudin-4 is reported to be highly expressed in gastric intestinal-type adenocarcinoma and several previous studies have shown that claudin-4 is involved in gastric cancer (21–23). For example, it has been previously reported that *Helicobacter pylori* has the ability to increase paracellular permeability by occludin, claudin-4 and claudin-5 (24). It has also been shown that caudal type homeobox 2 (Cdx2) is important in the regulation of intestinal claudin expression, not only in gastric mucosa with intestinal metaplasia, but also in gastric carcinoma (25). Furthermore, the involvement of specific claudin factors in Epstein-Barr virus-associated gastric cancer (26) and claudin-18 in signet ring cell cancer has previously been shown (27). In order to further clarify the mechanism of malignant transformation of gastric HPs, the present study analyzed four cases of cancer-bearing HPs using immunohistochemistry.

## Materials and methods

### Patients and specimens

In total, four patients with gastric polyps in the body of the stomach (three cases), antrum (two cases) and residual stomach (one case) were treated at the Department of Diagnostic Pathology, Graduate School of Medicine and Pharmaceutical Sciencies, University of Toyama, (Toyama, Japan), the Department of Surgical and Molecular Pathology, Dokkyo Medical University School of Medicine, (Tochigi, Japan) and the Department of Pathology, Ibaraki Preifectural Central Hospital (Ibaraki, Japan). Of these cases, four underwent endoscopic resection of the lesions and two cases underwent surgical removal of the lesions. The criteria for HPs was defined as hyperplastic foveolar epithelium without atypia and neoplastic epithelium was classified according to the Vienna classification (28). All patients provided written informed consent and the present study was approved by the ethics committee of our institute.

### Immunohistochemistry

Routinely-processed, formalin-fixed and paraffin-embedded tissue blocks were selected and 5-μm serial sections were prepared from the cut surface of the blocks. The antibodies, MUC1 (clone Ma695), MUC2 (clone Ccp58), MUC5AC (clone CLH2), MUC6 (clone CLH5; 1:200 dilution; Novocastra, Milton Keynes, UK), claudin-3 (polyclonal), claudin-4 (polyclonal), claudin-18 (polyclonal; 1:50 dilution; Zymed Laboratories, South San Franscisco, CA, USA), Cdx2 (clone CDX2–88; 1:500 dilution; BioGenex Laboratories, Freemont, CA, USA), p53 (clone DO-7; 1:100 dilution; Dako, Carpinteria, CA, USA) and Ki-67 (clone MIB-1; 1:100 dilution; Immunotech, Marseille, France) were used. Immunoperoxidase reactions were performed using the Ventana BenchMark^®^ LM automated immunostainer (Ventana Medical Systems, Tucson, AZ, USA) according to the manufacturer’s instructions. All cases were reviewed by two investigators, who arrived at a consensus on the pathological diagnoses and the assessment of immunoreactivity.

### Statistical analysis

Statistical analysis was performed using Student’s t-test for the comparisons of the p53 and Ki-67 labeling indices between each component. P<0.05 was considered to indicate a statistically significant difference.

## Results

### Clinicopathological observations

**Figure 1:**
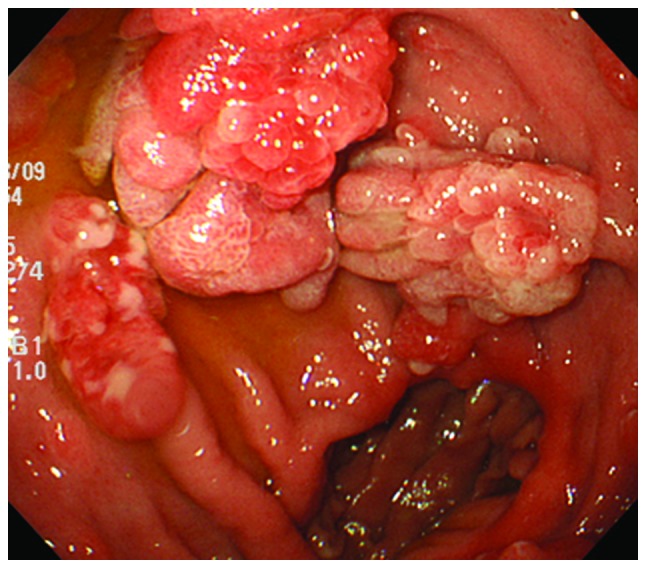
Endoscopic observations. Multiple polyps were shown to be scattered throughout the residual stomach in case 1.

The patients consisted of four males and two females, ranging in age between 65 and 78 years (mean age, 70 years). Endoscopic assessment revealed solitary (four cases) and multiple (two cases) pedunculated polyps in the gastric mucosa (Fig. 1). The patient characteristics are summarized in Table I.

### Pathological observations

**Figure 2:**
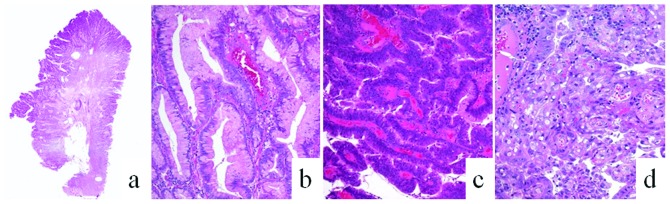
Histological observations of resected specimen of case 1 (H&E staining). (a) Low power view (magnification, ×4) of the carcinomatous component in a gastric hyperplastic polyp, consisting of (b) hyperplastic foveolar (magnification, ×10), (c) dysplasia (magnification, ×10) and (d) differentiated adenocarcinoma (magnification, ×10).

The resected polyps ranged in size between 10 and 40 mm in longitudinal diameter (mean size, 26 mm). All polyps were regionally composed of intermingled components of plural lesions in varied proportions. The lesions were basically composed of hyperplastic foveolar epithelium and intramucosal neoplasia, the latter of which was categorized as dysplasia and adenocarcinoma. The carcinomatous components were mainly differentiated as tubular adenocarcinoma. Although the border between each component was distinct, the transitional zone was relatively undefined (Fig. 2).

### Immunohistochemical observations

**Figure 3:**
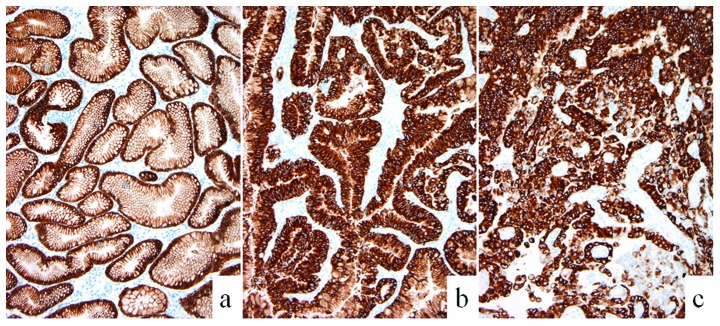
Immunohistochemical observations for MUC5AC in case 3: (a) foveolar hyperplasia, (b) dysplasia and (c) adenocarcinoma. Magnification, ×10.

**Figure 4:**
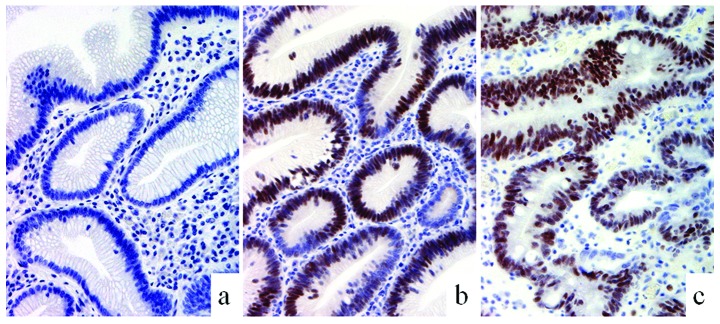
Immunohistochemical observations for caudal type homeobox 2 in case 4: (a) foveolar hyperplasia, (b) dysplasia and (c) adenocarcinoma. Magnification, ×10.

**Figure 5:**
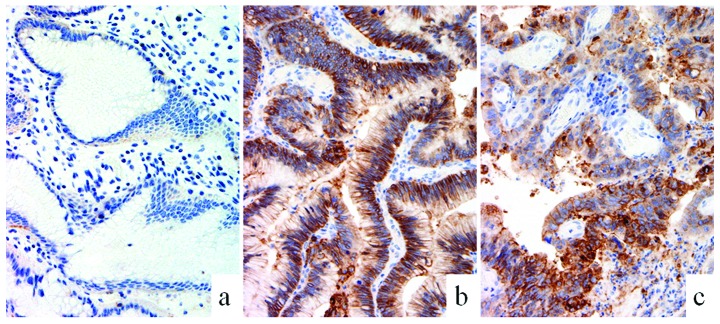
Immunohistochemical observations for claudin-4 in case 1: (a) foveolar hyperplasia, (b) dysplasia and (c) adenocarcinoma. Magnification, ×10.

The present immunohistochemical study found that MUC5AC was immunopositive in the hyperplastic, dysplastic and carcinoma regions of the polyps; while this marker was detected in the hyperplastic and dysplastic areas only, but not in the carcinomatous component, in one case. These observations suggested that the lesions were mostly of the gastric mucin type (Fig. 3). Furthermore, MUC2 expression was not observed in any of the specimens and goblet cells were also undetected, thus, the lesions were unlikely to be of the intestinal mucin type. Immunoreactivity for MUC1 in three cases and MUC6 in all cases were negative. Expression of Cdx2 in the nucleus of the cells of the intestinal epithelium was found in the dysplastic and carcinomatous components of all cases with the exception of one case (Fig. 4). The tight junction factor, claudin-3, was completely absent in the hyperplasia area, but was immunopositive in the dysplastic and carcinomatous components. By contrast, expression of claudin-4 was observed in the dysplastic and carcinomatous component of all cases (Fig. 5). In addition, expression of claudin-18 was observed in the hyperplastic, dysplastic and cancer components of all cases. The frequency of cells with abnormal nuclear accumulation of p53 was 12% in the hyperplastic area in one case while that of the other cases was <5%. By contrast, the frequencies ranged between 85–90% (mean, 88%) and 24–80% (mean, 64%) in the cancerous and dysplastic components, respectively. The percentage of Ki-67-positive cells was 10–25% (mean, 15%) in the hyperplastic areas, 55–70% (mean, 62%) in the dysplastic regions and 80–90% (mean, 84%) in the carcinomas. A statistically significant difference was observed between hyperplasia and each neoplastic (dysplasia and carcinoma) component (P<0.01). The immunohistochemical observations are summarized in Table II.

## Discussion

The current study immunohistochemically analyzed polyp samples from six patients that all exhibited regions of hyperplasia, dysplasia and carcinoma. From these observations, it was suggested that the malignant transformation of gastric HPs may occur by multistep carcinogenesis and these neoplastic cells may acquire various phenotypes during this process. As described in previous studies, a large polyp size is considered a risk factor for malignancy or may be a sign of malignant transformation (5,29). In the present study, all the polyp samples exceeded 10 mm in diameter (mean diameter, 26 mm) and consisted of hyperplasia, dysplasia and carcinoma, thus, consistent with the observations of previous studies. All the carcinomatous components were essentially composed of well-differentiated adenocarcinomas, and poorly differentiated adenocarcinomas were not included in the current series. Although certain cases of poorly differentiated adenocarcinomas in HPs have been previously reported, differentiated adenocarcinoma is considered to be the most common histological type of carcinoma based on previous large-scale studies (5,30).

With regard to the phenotype of the HPs, it appeared to be of the gastric type, since MUC5AC was detected not only in all the hyperplastic components, but also in all the dysplastic and carcinomatous lesions. Thus, it was suggested that the neoplastic lesions in all cases of the current series retained the gastric phenotype even following malignant transformation. However, previous studies have presented arguments with regard to the intestinalization of gastric epithelial neoplasia; although, the evidence is inconclusive, largely due to the reason that accurate and specific markers of intestinalization are not yet available (5,7,14,30). However, since MUC2 expression is positive in goblet cells and CD10 is detected in the brush border, these markers may be important for the acquisition of the intestinalization phenotype in neoplastic cells and, thus, may be good candidate markers for this process (16–18). Cdx2 is a transcription factor involved in the differentiation of the intestinal epithelium and, by immunohistochemistry, it was revealed that this protein was expressed in the nucleus (31). The expression of Cdx2 often parallels that of MUC2, but these two proteins are not necessarily positively correlated. Cdx2 appears to be expressed at the stage of the precursory intestinal epithelium (31); therefore, this protein may be more useful as a marker to detect the early stages of the intestinalization phenotype.

In the current study, claudin-4 was immunopositive in regions of dysplasia and/or carcinoma in all cases. It has previously been reported that claudin-4 is expressed only in cancer, whereas it is not expressed in the normal foveolar epithelium (21–23). The present study also obtained similar observations for claudin-3 in five of the cases. According to a previous study by Shinozaki *et al*, claudin-3 appears to be expressed in intestinal metaplasia in a similar expression pattern as that of MUC2 and CD10 (26). However, claudin-3 appears to be expressed during the early stage of intestinalization, similar to Cdx2 expression in the present study. By contrast, the expression pattern of claudin-18 was similar to that of MUC5AC. These results indicated that the neoplastic lesions show an extremely similar phenotype to gastric foveolar epithelium (26).

p53 protein is considered to be one of the most important gene products during the carcinogenesis of various malignancies, including gastric cancer (32). In the current study, nuclear accumulation of p53 was highly detected in the neoplastic lesions (dysplasia and carcinoma), but was absent or extremely low in the areas of hyperplasia, with the exception of one case. Furthermore, it was found that the Ki-67 labeling index gradually increased from hyperplasia to dysplasia to carcinoma. These observations provided evidence to support the importance of the hyperplasia-dysplasia-carcinoma sequence during malignant transformation of HPs (8).

In conclusion, during the malignant transformation of gastric HPs, cancer may spontaneously occur in the lesion through multistep carcinogenesis, such as the hyperplasia-adenoma (dysplasia)-adenocarcinoma sequence. The neoplastic cells may acquire various phenotypes during this process. Since it appears that several gastric and intestinal phenotypes are admixed in a complex manner within these polyps, the malignant transformation of the cells may not necessarily undergo simple differentiation.

## Figures and Tables

**Table I:** List of patient characteristics.

	Patient					
	
Variable	1	2	3	4	5	6
Age, years	69	78	65	68	75	66
Gender	M	F	M	M	F	M
Location	RS	B	A	B	A	B
Lesion	M	S	S	S	S	M
Size, cm	4.0	2.5	3.0	1.0	2.5	2.6

a RS, residual stomach; B, body; A, antrum.

b M, multiple; S, solitary. M, male, F, female.

**Table II:** Results of immunohistochemical analysis.

	Case 1			Case 2			Case 3			Case 4			Case 5			Case 6		
						
Markers	H	D	C	H	D	C	H	D	C	H	D	C	H	D	C	H	D	C
MUC1	−	−	−	−	−	−	−	+	+	−	+	+	−	−	−	−	+	+
MUC2	−	−	−	−	−	−	−	−	−	−	−	−	−	−	−	−	−	−
MUC5AC	++	++	++	++	++	++	++	++	−	++	++	++	++	++	++	++	++	++
MUC6	−	−	−	−	−	−	−	−	−	−	−	−	−	−	−	−	−	−
Cdx2	−	−	−	−	+	++	+	++	++	−	++	++	−	++	++	−	++	++
Claudin-3	−	+	+	−	+	+	−	−	+	−	−	−	−	−	+	−	−	+
Claudin-4	++	++	++	−	++	++	−	++	++	++	++	++	++	++	++	++	++	++
Claudin-18	++	++	++	++	++	++	++	++	++	++	++	++	++	++	++	++	++	++
p53, %	<5	24	85	12	80	90	<5	80	90	<5	60	90	<5	75	85	<5	65	90

H, hyperplasia; D, dysplasia; C, carcinoma; Cdx2, caudal type homeobox 2.
